# Osgood-schlatter disease: risk of a disease deemed banal

**DOI:** 10.11604/pamj.2017.28.56.13185

**Published:** 2017-09-21

**Authors:** Mustafa Nkaoui, El Mehdi El Alouani

**Affiliations:** 1 Service de Chirurgie Orthopédique et de Traumatologie, CHU Ibn Sina, Université Mohammed V Souissi, Rabat, Maroc

**Keywords:** Osgood-schlatter, avulsion, anterior tibial tuberosity

## Image in medicine

Osgood-schlatter disease, non-articular osteochondrosis, is characterized by pain of anterior tibial tuberosity (TTA), which mainly affects the growing adolescent sportsman. It corresponds to “chronic lesions, due to microtrauma of repeated tractions on the area of insertion of the patellar tendon at the level of the anterior tibial tuberosity”. This condition remains benign if the young patient rests well, the only treatment proven effective to avoid complications such as the removal of the anterior tibial tuberosity. The case reported in this article illustrates this complication. He is a 15-year-old boy, followed for Osgood-Schlatter disease, who did not respect the advice given by his attending physician; and who was the victim of an accident during a soccer match: brutal contraction of the quadriceps during an upset extension of the left knee, causing pain and total functional impotence. The examination showed a large oedematous knee with pain on palpation of TTA, with a deficit of active extension of the knee (A). Standard radiographs of the left knee showed avulsion of the anterior tibial tuberosity (B). The treatment was surgical with reduction of the fragment and osteosynthesis by two cortical screws through the median approach (C). With a follow-up of 6 months, the knee mobility was normal and the fracture consolidated. Osgood-Schlatter disease, a pathology of growth, is generally of good prognosis, resolving spontaneously with skeletal maturity. However, if the young athlete does not change his practice, he may seriously aggravate it (removal of the TTA requiring orthopedic or even surgical treatment) and therefore with much more serious consequences for its sporting recovery.

**Figure 1:**
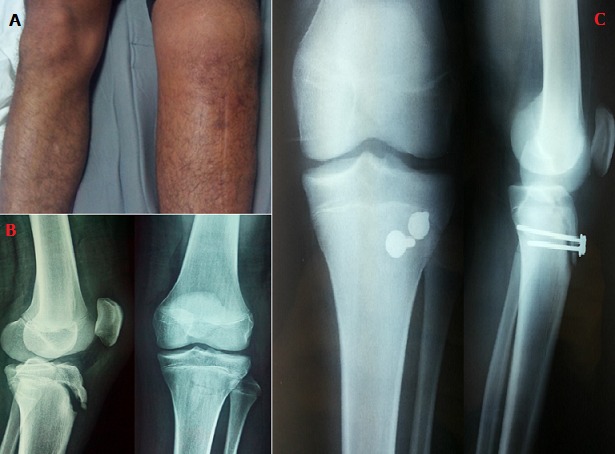
(A) clinical aspect of an anterior tibial tuberosity removal: large left knee oedematous + deficit of active extension of the same knee; (B) standard radiography of the left knee: avulsion of the anterior tibial tuberosity; (C) radiography of postoperative control: reduction + osteosynthesis by two cortical screws

